# Supercritical CO_2_ Extract from Microalga *Tetradesmus obliquus*: The Effect of High-Pressure Pre-Treatment

**DOI:** 10.3390/molecules27123883

**Published:** 2022-06-17

**Authors:** Jelena Vladić, Igor Jerković, Sanja Radman, Jelena Molnar Jazić, Alice Ferreira, Snežana Maletić, Luisa Gouveia

**Affiliations:** 1 Faculty of Technology, University of Novi Sad, Bulevar Cara Lazara 1, 21102 Novi Sad, Serbia; vladicjelena@gmail.com or; 2 Faculty of Chemistry and Technology, University of Split, Ruđera Boškovića 35, 21000 Split, Croatia; sradman@ktf-split.hr; 3 Faculty of Sciences, University of Novi Sad, Trg Dositeja Obradovića 3, 21102 Novi Sad, Serbia; jelena.molnar@dh.uns.ac.rs (J.M.J.); snezana.maletic@dh.uns.ac.rs (S.M.); 4 LNEG, National Laboratory of Energy and Geology I.P., Bioenergy and Biorefineries Unit, Paço Lumiar 22, 1649-038 Lisbon, Portugal; alice.ferreira@lneg.pt; 5 GreenCoLab—Green Ocean Technologies and Products Collaborative Laboratory, Centro de Ciências do Mar do Algarve, Universidade do Algarve, Campus Gambelas, Edifício 7, 8005-139 Faro, Portugal

**Keywords:** *Tetradesmus obliquus*, supercritical carbon dioxide, microalga, high pressure, green extraction, UHPLC-ESI-HRMS

## Abstract

High-pressure pre-treatment followed by supercritical carbon dioxide (ScCO_2_) extraction (300 bar, 40 °C) was applied for the attainment of the lipophilic fraction of microalga *Tetradesmus obliquus*. The chemical profile of supercritical extracts of *T. obliquus* was analyzed by ultra-high-performance liquid chromatography–high-resolution mass spectrometry with electrospray ionization (UHPLC-ESI-HRMS). Moreover, the impact of ScCO_2_ on the microbiological and metal profile of the biomass was monitored. The application of the pre-treatment increased the extraction yield approximately three-fold compared to the control. In the obtained extracts (control and pre-treated extracts), the identified components belonged to triacylglyceroles, fatty acid derivatives, diacylglycerophosphocholines and diacylglycerophosphoserines, pigments, terpenes, and steroids. Triacylglycerols (65%) were the most dominant group of compounds in the control extract. The pre-treatment decreased the percentage of triacylglycerols to 2%, while the abundance of fatty acid derivatives was significantly increased (82%). In addition, the pre-treatment led to an increase in the percentages of carotenoids, terpenoids, and steroids. Furthermore, it was determined that ScCO_2_ extraction reduced the number of microorganisms in the biomass. Considering its microbiological and metal profiles, the biomass after ScCO_2_ can potentially be used as a safe and important source of organic compounds.

## 1. Introduction

Microalgae represent a topic of interest owing to their numerous benefits. Namely, they are characterized by undemanding cultivation and fast growth, prevailing in natural habitats such as freshwater, brackish and seawater, and marginal lands. Additionally, they can be obtained from wastewater treatment, reducing cultivation costs and environmental impacts, and resulting in additional benefits such as clean water and valuable biomass. Finally, the biomass of these photosynthetic organisms contains different compounds, which can be applied in the production of biofuels, agricultural products, animal feed, food, pharmaceutics, and cosmetics [1]. 

The use of microalgae as a source of bioactive compounds in the food, feed, cosmetic, and pharmaceutical industries brings important advantages, including the use of low-cost and renewable natural resources instead of organic synthesis for obtaining high-value compounds and products, thus reducing the negative environmental impact. In addition, using natural compounds has become a highly demanded aspect that can influence the acceptance of products in the market due to the consumers’ increased health and environmental awareness. Moreover, the use of microalgae to satisfy the growing market demand for compounds such as fatty acids and carotenoids represents a significant contribution to the sustainable production and economy [2,3]. 

*Tetradesmus obliquus* is a freshwater microalga, suitable for large-scale production because of its robustness, undemanding cultivation, and rapid growth [4]. *T. obliquus* represents an excellent feedstock for obtaining different compounds in a biorefinery platform, such as bioH_2_ (by dark fermentation), bio-oil, bio-char and biogas (by pyrolysis), phenols and flavonoids (through subcritical water extraction), and biostimulants for several seeds’ germination [5]. Aside from the significant presence of lipids, this microalga possesses important biomolecules such as carotenoids. The isolation of these compounds is usually achieved by extraction with organic solvents [3,6]. However, using organic solvents reduces the safety and limits the application of the obtained products. As a green alternative method to conventional organic solvent extraction, supercritical carbon dioxide (ScCO_2_) extraction has been established. Apart from the safe solvent, it provides a reduced extraction time compared to conventional extractions such as Soxhlet, with the possibility of adjusting products’ features by process parameter manipulation. Moreover, ScCO_2_ extraction allows the attainment of clean extracts without solvent residue [7].

Solana et al. confirmed the advantages of ScCO_2_ extraction of *T. obliquus* over the less selective Soxhlet extraction [8]. The addition of 5% ethanol as an entrainer to ScCO_2_ extraction (60 °C, 300 bar, 0.4 kg/h) provided the highest extraction yield. Moreover, using 7.7% (*v*/*v*) ethanol as a co-solvent in ScCO_2_ extraction increased the carotenoid and chlorophyll extraction [9]. Furthermore, *T. obliquus* biomass mixed with diatomaceous earth (1:10 *w*/*w*) was extracted by using the following parameters: pressure 100–350 bar, temperature 40–60 °C, and co-solvents methanol (5% *v*/*v*) and limonene (5% *v*/*v*) [10]. However, the application of the co-solvents aggravated the implementation of the process at industrial level due to safety rules and regulations. Therefore, Lorenzen et al. investigated the ScCO_2_ extraction of lipids of *T. obliquus* and *Scenedesmus obtusiusculus* using an industrial pilot plant [11]. They showed that the application of pressure of 120 bar, a temperature of 20 °C, and a CO_2_ to biomass ratio of 100 provided a high lipid extraction yield, achieving approximately 92% *w*/*w* of total lipids. ScCO_2_ extraction fulfills the green extraction principles; however, after the extraction, a material remains that still possesses different bioactive compounds. Rational utilization of waste would represent a solution that can be relevant for the industry and beneficial for the environment. In our previous work, the ScCO_2_ extraction of *T. obliquus* was conducted at 300 bar and 40 °C [12]. Due to the very low yield, the obtained extract was not analyzed. It was concluded that the microalgal material needed to be pre-treated to improve its extraction efficiency. Furthermore, the biomass used for the ScCO_2_ extraction was further used in ultrasound-assisted, microwave-assisted, and subcritical water extractions. Moreover, for comparison, ScCO_2_-untreated biomass was used in the aforementioned extractions. It was established that by using the exhausted biomass (after ScCO_2_ extraction), higher extraction yields were obtained in all extractions (ultrasound-assisted, microwave-assisted, and subcritical water extraction). Hence, it was concluded that ScCO_2_ extraction can function as a pre-treatment that contributes to the more efficient exploitation of *T. obliquus* biomass. During the extraction with compressed CO_2_, the material is exposed to high pressure; therefore, changes in and disturbance of the material structures can occur. In this way, material resistance can be reduced, facilitating solvent penetration and contact with the target components. 

In addition, ScCO_2_ was investigated as a treatment for the inactivation of microorganisms. Due to mild pressure and temperature conditions, it can represent an alternative to conventional sterilization procedures that apply high temperatures and long process times, which implies more energy. Moreover, ScCO_2_ does not involve the addition of other additives and can be removed from the material easily [13].

Having in mind that microalgae are usually grown in outdoor open ponds, their biomass can be contaminated and can contain a significant number of microorganisms [12]. The application of ScCO_2_ to neutralize microorganisms in the microalgae biomass has not been investigated previously. 

Therefore, the goal of this study was to investigate the possibility of applying high-pressure pre-treatment followed by ScCO_2_ to (1) increase the efficiency and yield of the extraction and (2) neutralize microorganisms present in the biomass. The ScCO_2_ extracts of *T. obliquus* were analyzed via ultra-high-performance liquid chromatography–high-resolution mass spectrometry (UHPLC-ESI-HRMS) for the first time. Moreover, the impact of the applied green solvent ScCO_2_ on the biomass was evaluated by monitoring the microbiological and metal profiles of the biomass. 

## 2. Results and Discussion

### 2.1. Supercritical Carbon Dioxide (ScCO_2_) Extraction

Microalgae are characterized by a thick cell wall that impedes the extraction of bioactive components. Therefore, different pre-treatments were explored, with the goal to improve the release of lipid compounds from the microalgae matrix. Due to the structure of the microalgae cell wall, which consists of carbohydrates and glycoproteins, enzymatic pre-treatments showed promising effects [14]. However, these treatments require a long time and several steps, involving additional costs related to the production of enzymes [15]. Ansari et al. applied microwave, sonication, autoclaving, and osmotic shock as treatments for the disruption of *S. obliquus* biomass before extraction with organic solvents (chloroform–methanol; 2:1, *v*/*v*) [16]. They established that the highest lipid yield could be achieved after freeze drying followed by microwave digestion. For the disruption treatment of *S. obliquus*, *Chlorella vulgaris*, and *Botryococcus* sp., autoclave, microwave, sonication, bead beating, and 10% NaCl treatments were applied. The microwave treatment was established as the most optimal disruption pre-treatment [17]. *Nannochloropsis* sp. biomass was subjected to incubation at 37 °C for 15 h before treatment with high-pressure homogenization at 1200 ± 100 bar, followed by lipid extraction with hexane [18]. For the disruption of *S. obliquus*, pressure homogenization (150 bar) was applied, followed by lipid extraction with hexane and ethyl acetate and dry extractions using hexane [19]. A pulsed electric field was applied as a pre-treatment of *Ankistrodesmus falcatus* biomass, resulting in a significant improvement in the extraction of lipids [20]. 

Therefore, pre-treatments can significantly improve microalgae extraction; however, they include additional equipment, which elevates the production costs. If a pre-treatment includes moisturizing the material, it is necessary to include the drying process for the purpose of ScCO_2_ extraction. The additional steps complicate and increase the costs of the process, increasing the possibility of compound loss, particularly volatile ones, and contamination. For these reasons, to improve the exploitation of microalgal material and increase the ScCO_2_ extraction yield, high-pressure pre-treatment followed by decompression was applied. The pre-treatment was conducted in the same extraction cell where the extraction was conducted. In this way, time and labor were minimalized. Moreover, a control extraction was conducted without the pre-treatment. 

**Figure 1:**
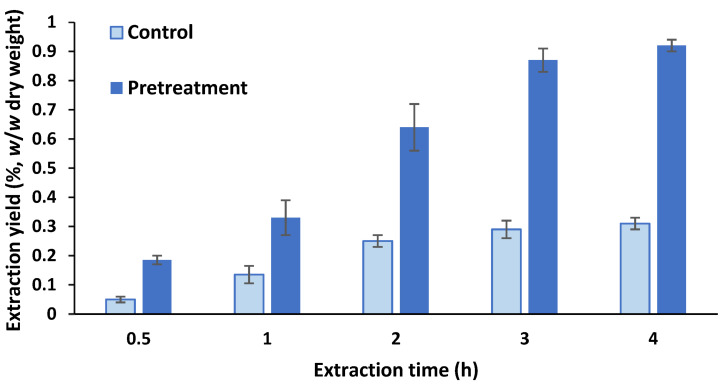
Extraction yield expressed as % (*w*/*w* dry weight). Kinetics of supercritical CO_2_ extraction (pressure 300 bar, temperature 40 °C) of *Tetradesmus obliquus* biomass, described in Section 3.2 in detail.

The extraction yield of the control ScCO_2_ extraction was 0.31 ± 0.02% *w*/*w* dry weight (extraction time 4 h) (Figure 1 and Table S1). Application of high-pressure pre-treatment followed by decompression significantly accelerated and improved the ScCO_2_ extraction; therefore, the extraction yield of the control extraction was surpassed after 1 h of extraction. By monitoring the extraction kinetics, it was determined that one third of the lipid fraction was extracted after 1 h, whereas, after 2 h of extraction, more than 60% was achieved. Further prolongation of the extraction decreased the percentage of the extracted lipid fraction. The total extraction yield achieved after the pre-treatment was approximately three-fold higher (0.92 ± 0.02% *w*/*w* dry weight) than the control (0.31 ± 0.02% *w*/*w* dry weight). 

The static exposure to compressed CO_2_ softened and weakened the cell structures. Since the material was saturated with CO_2_, by applying decompression, a pressure gradient was formed. Due to decompression, pressure on the cellular structure decreased and the intraparticle resistance was reduced. Therefore, after pre-treatment, the cell structure became more permeable, allowing easier penetration and contact between ScCO_2_ and the compounds, facilitating their release. High-pressure pre-treatment with decompression was previously shown to be adequate for the disruption of the glandular structures of *Origanum virens* [21] and *Hypericum perforatum* [22]. Moreover, a higher extraction yield of *Satureja montana* was obtained after exposure of the material to compressed CO_2_ [23]. 

The total lipid content determined via Soxhlet extraction as the reference method after 6 h extraction with methylene chloride was 9.1% (*w*/*w* dry weight). This significantly higher yield of total fats pointed to a potential need for further investigation of the pretreatments with the aim of increasing the extraction yield. However, Soxhlet extraction is characterized by health and ecological unsafety and extraction non-selectivity, whereas ScCO_2_ can be used safely for the pre-treatment and achievement of selective extraction. 

Apart from improving the extraction yield, an important aspect of the application of the pre-treatment represents its impact on the quality of the extract. Therefore, the control extract and the extract obtained after the pre-treatment were analyzed by using UHPLC-ESI(+)-HRMS) to establish the pre-treatment’s influence on the chemical profile. 

UHPLC-ESI(+)-HRMS analysis revealed the presence of valuable components such as pigments, lipids, and terpenes, indicating that the investigated ScCO_2_ extracts represent an important source of different bioactive compounds. In the control extract, 27 compounds were detected, whereas in the extract obtained with the pre-treatment, 25 compounds were detected (Table 1). The extracts were rich in lipids including triacylglyceroles, fatty acid derivatives, diacylglycerophosphocholines, and diacylglycerophosphoserines. A similar lipid profile was also found in *Chlorella vulgaris* [24].

**Figure 2:**
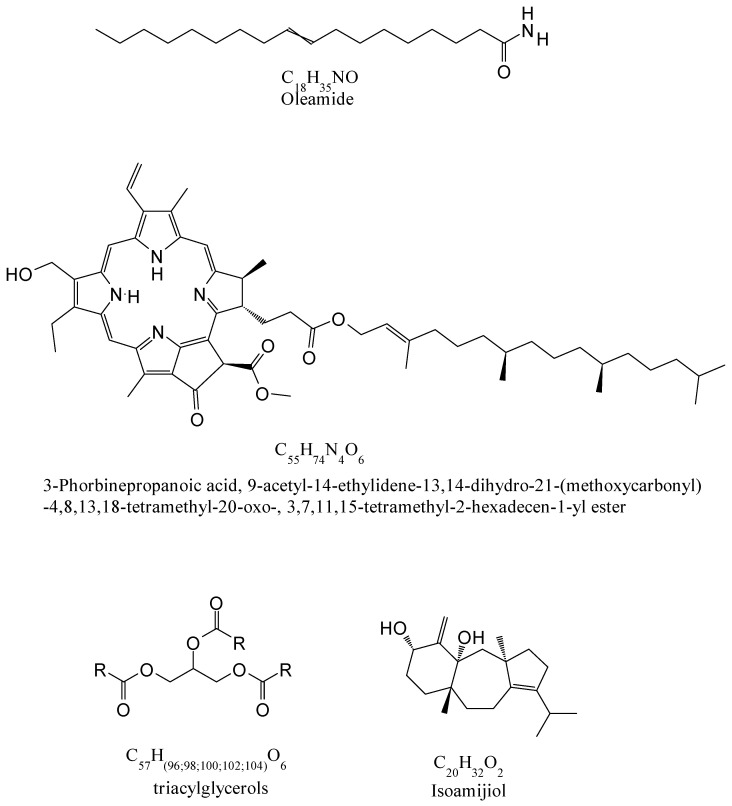
Chemical structures of most abundant compounds analyzed by UHPLC-ESI(+)-HRMS.

Furthermore, the control extract had a higher abundance of lipids and the most dominant compounds belonged to triacylglycerols (65%), followed by fatty acid derivatives (30%) (Figure 2). The pre-treatment extract, on the other hand, was mainly composed of fatty acid derivatives (82%) and only 2% of triacylglycerols. As triacylglycerols represent a result of the esterification of fatty acids and glycerol, it is possible that their conversion occurred due to exposure to pre-treatment stress. Producing biodiesel in noncatalytic supercritical processes with alcohols was previously suggested as an alternative to conventional transesterification procedures [25]. Moreover, it was established that using CO_2_ as a co-solvent can improve the supercritical transesterification [26].

Long-chain triacylglycerols were identified in the extracts, among which triacylglycerol 54:5 was the most abundant. In the group of fatty acid derivatives, oleamide, an amide of oleic acid, was the most abundant. In vivo studies suggested that oleamide exhibits significant anti-inflammatory activity and can contribute to the prevention of Alzheimer’s disease [27,28].

Pheophytin *a* is a derivative of chlorophyl *a* and was detected in both *T. obliquus* extracts at a low percentage. Its presence in *T. obliquus* extracts was also determined by Gilbert-López et al. [29]. Shailaja et al. suggested that it could be beneficial in lung cancer cell treatment, since pheophytin *a* isolated from seagrass impacted metastatic alveolar cancer cells, causing apoptosis-induced death [30]. Additionally, it was indicated that pheophytin *a* from *Lonicera hypoglauca* Miq. could be used for the treatment of hepatitis C virus because of its anti-NS3 protease activity [31]. Anti-inflammatory [32] and anticarcinogenic properties [33] of pheophytin *a* from *Enteromorpha prolifera* were determined. Moreover, pheophytin *a* from brown alga *Sargassum fulvellum* exhibited neuroprotective properties by promoting neurite outgrowth [34].

Although the presence of carotenoids in the extracts was low, in the extract obtained from the pre-treated biomass (1%), it was two-fold higher compared to the control (0.5%). The improved extraction of carotenoids from *Botryococcus braunii* after CO_2_ rapid depressurization pre-treatment was also obtained by Uquiche et al. [35]. The high-pressure pre-treatment caused pressure on the microalgae cells without the generation of heat, which usually occurs in mechanical pre-treatments such as ultrasound and microwave. Therefore, the cells became more porous, and the release of compounds was facilitated without the degradation of temperature-sensitive compounds such as carotenoids. The mild temperature of ScCO_2_ used in the pre-treatment and extraction allowed for the increased release of carotenoids without compromising their structure [35]. In addition, carotenoids are unstable and susceptible to degradation due to exposure to light, heat, oxygen, and other factors. ScCO_2_ extraction includes the application of a mild temperature and the absence of oxygen and light, making it adequate for carotenoids’ extraction [36].

In the control extract, among carotenoids, phytoene epoxide, vaucheriaxanthin, and myxol 2’-fucoside were equally present (approximately 32%), while there was 4% echinenone. In the pre-treated sample, the most dominant carotenoid was phytoene epoxide with more than 50%, followed by vaucheriaxanthin (27.52%), echinenone (12.31%), and myxol 2’-fucoside (5.86%). The carotenoids identified in the extracts belong to the group of xanthophylls, which are oxygen-containing carotenoids. It is assumed that xanthophylls exert greater bioaccessibility compared to carotenes, which is attributed to the presence of hydroxyl groups that can regulate and improve solubility [37].

Because of their exceptional antioxidant potential, the application of echinenone and myxol 2’-fucoside (myxoxanthophyll) in pharmaceutical, food, cosmetic, and animal nutrition products is recommended [38]. Moreover, carotenoid glycoside myxoxanthophyll possesses anti-hyperglycemic potential [39]. Ketocarotenoid echinenone is characteristic of cyanobacteria. This ketocarotenoid was detected in a higher percentage in the pre-treated extract of *T. obliquus*. Echinenone could be obtained as a by-product of astaxanthin production with microorganisms [40] and as a derivate of *β*-carotene through chemical processes [41]. It is a more potent antioxidant than astaxanthin [42] and its transformation to vitamin A in the liver contributes to echinenone’s medicinal importance [40].

Finally, compounds that belong to the groups of terpenoids and steroids were detected in supercritical extracts (13.82% in the pre-treated extract and 2.48% in the control extract). Monoterpenoid lactone loliolide was identified in both extracts. Loliolide was previously isolated from different seaweeds, such as *Codium tomentosum* [43], *Sargassum horneri* [44], and *Undaria pinnatifida* [45]. It is considered to possess antioxidant and anticancer activity. In addition, it is suggested that loliolide could be applied in the development of new neuroprotective therapeutics for Parkinson’s disease, due to its antioxidant, anti-inflammatory, and neuroprotective mechanisms [43]. Moreover, among representatives of terpenoids, isoamijiol and isoamijiol oxidation products were identified. These terpenoids were previously identified in macroalgae *Fucus virsoides* [46] and *Codium adhaerens* C. Agardh 1822 [47], as well as in brown alga *Dictyota linearis* [48]. Moreover, (3β)-3-Hydroxystigmast-5-en-7-one from the stigmastanes class was identified in the extracts.

### 2.2. Microbiological Profile

Natural materials such as plants and microalgae can have a significant number of microbiological contaminants originating from the environment. Moreover, inadequate storage or high moisture levels can cause increased levels of contaminants. The presence of enterobacteria and *Escherichia coli* can indicate fecal contamination. Moreover, molds and yeasts can decrease the shelf life and quality of products [49].

Since contaminants can limit the application of products, it is important to determine their presence [49]. Previously, it was confirmed that ScCO_2_ can be applied for the sterilization of different materials [50,51]. Due to the possibility of inactivating microorganisms at low temperatures, ScCO_2_ is adequate for thermolabile materials (Table 2). This property is attributed to the impact of ScCO_2_ on the bacteria cell wall, where the implosion of the cell wall and accelerated penetration of the ScCO_2_ in the cells occur due to high pressure [52].

In the *T. obliquus* initial biomass, the presence of microorganisms 910 × 10^4^ cfu/g, Enterobacteriaceae 49 × 10^3^ cfu/g, *Escherichia coli* < 40 cfu/g, and spores of anaerobic bacteria 240 × 10^2^ cfu/g was determined. By analyzing the same microbiological parameters in the ScCO_2_-spent biomass, it was established that the total number of microorganisms and the presence of spores of anaerobic bacteria were reduced approximately three-fold and Entererobacteriacae were reduced below 10 cfu/g. Therefore, it can be confirmed that the application of ScCO_2_ can, apart from the extraction of bioactive compounds, reduce the presence of contaminants in the biomass. The microbiologically safe biomass represents a precondition for further rational application and valorization. The advantage of the ScCO_2_ treatment is that the extraction and purification of the biomass occur at the same time and within the same extraction unit, which is industrially feasible.

In the absence of other regulations, the microbiological profile of the biomasses was evaluated and compared with the values regulated by the European Pharmacopoeia for herbal medicinal products consisting solely of one or more herbal drugs (whole, reduced, or powdered). The allowed presence of molds and yeasts in herbal medicinal products is 10^5^ and 10^4^ cfu/g, when boiling water is previously added or not, respectively. According to these parameters, the levels of molds and yeasts in both biomasses were below the prescribed values. Furthermore, the presence of *Escherichia coli* was under the allowed value (10^3^ cfu/g). The microbial limit for Enterobacteria and other Gram-negative bacteria is 10^3^ cfu/g [49]. Therefore, the initial biomass had these contaminants above the allowed values. However, after ScCO_2_ extraction, these values were reduced and under the allowed values prescribed for herbal medicinal products.

Additionally, the ScCO_2_ treatment reduced the content of moisture in the material, since the initial value was 4.67%, whereas, after the treatment, it was reduced to 2.8%. The reduction of moisture in the material inhibits microbial and enzymatic activities, secures product stability, and extends the shelf life. Moisture content below 10% is considered adequate for the long-term storage of microalgae [53,54].

### 2.3. Determination of Metal Content

The inadequate disposal of waste material represents an environmental risk. Hence, it is important to establish the characteristics of the waste and explore the possibility of its potential application or safe disposal. The significant number of organic compounds, such as aliphatic saturated and unsaturated hydrocarbons, alkylated hydrocarbons, ketones, phenols, and esters, in the biomass indicates that these materials have the potential to be used as a source of various bioactive compounds. These compounds can be used in pharmaceutical and cosmetic areas, and in agriculture as animal feed or a soil fertilizer or conditioner [12]. Despite the decrease in the number of microorganisms in the biomass with ScCO_2_, the presence of heavy metals can limit the application or safe disposal of these biomasses. For this reason, the level of metals was investigated in the initial biomass and the biomass after ScCO_2_ extraction (Table 3).

The concentration of metals in the initial biomass was comparable with the level of metals obtained in a previous study, except for Fe, which was lower in the present study (2510 mg/kg vs. 6170 mg/kg [12]). No significant changes in metal concentrations were found after the ScCO_2_ extraction between the initial biomass and the spent biomass. The result implies that ScCO_2_ does not extract heavy metals, producing extracts that are metal-free. The higher difference in the detected Fe concentration between initial and ScCO_2_-spent biomass samples could potentially be due to the higher measurement uncertainty for this analyte (30%).

According to the content of heavy metals, both the initial biomass and ScCO_2_-spent biomass can be used as animal feed (Directive 2002/32/EC) [55] and in agriculture as a conditioner and soil fertilizer (Directive 86/278/EEC) [56]. Fe and Mn, which were present in higher concentrations, are not toxic metals and, thus, are not regulated by these directives.

## 3. Materials and Methods

### 3.1. Microalgae Cultivation

*T. obliquus* (formerly known as *Scenedesmus obliquus*) (ACOI 204/07, ACOI Culture Collection, Coimbra University, Coimbra, Portugal) biomass was obtained from an outdoor raceway pond (4500 L) located at the LNEG’s Lumiar Campus in the city of Lisbon, on the western coast of Portugal (38°42′ N, 9°11′ W). The microalga was cultivated in Bristol medium at pH 7 in natural light/dark cycles (average radiation during daylight time was 382.8 W/m^2^—10.5 h daily; average air temperature was 16.9 °C (6.8–29.8 °C) for 55 days, from September to November). The biomass was collected through decantation prior to centrifugation at 10,000 rpm and 4 °C for 15 min. The biomass was dried at 80 °C until constant weight and stored at −18 °C for further studies [57].

### 3.2. Supercritical Carbon Dioxide Extraction

The extraction process was carried out in a laboratory-scale high-pressure extraction system (HPEP, NOVA, Swiss, Effertikon, Switzerland). The specifications of the ScCO_2_ system were as follows: CO_2_ gas cylinder, diaphragm-type compressor (with 1000 bar maximum pressure), extractor vessel with heating jacket (internal volume 200 mL, maximum operating pressure 700 bar), separator with cooling jacket (internal volume 200 mL and maximum operating pressure 250 bar), pressure control valve, temperature regulation system, and regulation valves. For the pre-treatment, the material (50 g) was exposed to ScCO_2_ (static) for 1 h, after which decompression was applied. To determine the most adequate pressure value for the pre-treatment, four different pressures were tested: 100, 200, 300, and 400 bar. Temperature was maintained at 40 °C to avoid the loss of thermosensitive compounds. After the pre-treatments, the extraction was conducted for 4 h at 300 bar and 40 °C, and a CO_2_ flow rate of 0.194 kg/h. The separator conditions were maintained constant at 15 bar and 23 °C. The pressures of 300 and 400 bar provided the highest extraction yield; however, due to lower pressure and costs, 300 bar was chosen for the pre-treatment. Control extraction was conducted under the same extraction conditions (4 h at 300 bar and 40 °C), without pre-treatment. After the extraction, the extracts were collected into glass vials and stored in a dark place at 4 °C until analysis. Moreover, the biomass after the ScCO_2_ extraction (ScCO_2_-spent) was further used for the analyses. The extraction yield was determined gravimetrically and calculated using the following equation:

(1) $$\mathrm{Extraction}\;\mathrm{yield}\;\left(\%\right)=\frac{\mathrm{mass}\;\mathrm{of}\;\mathrm{obtained}\;\mathrm{extract}\;\left(g\right)}{\mathrm{mass}\;\mathrm{of}\;\mathrm{feed}\;\mathrm{material}\;\left(g\right)}\times 100$$

### 3.3. Ultra-High-Performance Liquid Chromatography–High-Resolution Mass Spectrometry (UHPLC-ESI-HRMS) Analyses of CO_2_ Extract

The UPLC-HRMS analyses of the CO_2_ extract were performed using an ExionLC AD system (AB Sciex, Concord, ON, Canada) equipped with the ExionLC solvent delivery system, pump with degasser, column oven, autosampler, and controller, combined with a quadrupole-time-of-flight (Q-TOF) mass spectrometer, the TripleTOF 6600+ (AB Sciex, Concord, ON, Canada), with a Duospray ion source. The analytical column used for chromatographic separations was the Acquity UPLC BEH Phenyl-Hexyl, 2.1 mm 100 mm, particle size 1.7 m (Waters, Milford, MA, USA). The column oven temperature was set at 30 °C and the flow rate at 0.4 mL/min. The mobile phases were water (A) and acetonitrile (B), both containing 0.1% formic acid. After 0.6 min under isocratic condition with 2% of B, the elution program was applied as follows: 0.6–18.5 min (B linear gradient to 100%), 18.5–25 min (100% B). The injection volume was 4 L.

Mass spectrometry detection was conducted with positive electrospray ionization (ESI+). Tandem (MS/MS) mass spectra were recorded using the collision-induced dissociation (CID) in information-dependent acquisition mode for precursor ions with the signal intensities above the 200 counts per second (cps) threshold. The maximum number of precursor ions was set to 15. The ion source parameters were: curtain gas (nitrogen) pressure 30 psi, heater gas (air, gas 2) pressure 15 psi, nebulizing gas (air, gas 1) pressure 40 psi, ESI capillary voltage 5.5 kV, and the source temperature of 300 °C. The recording mass spectra parameters were: declustering potential 80 V, *m*/*z* range 100–1000 (MS) and 20–1000 (MS/MS), and accumulation time of 100 ms. The collision gas was nitrogen and the collision energy was set to 40 eV with a spread of 20 eV. The mass scale calibrations (MS and MS/MS modes) were done prior to each run of sample in an automatic regime using a Tuning Solution (AB Sciex, Concord, ON, Canada).

The data were processed using ACD/Spectrus Processor 2021.1.0 (ACD/Labs, Toronto, ON, Canada). The elemental compositions of the compounds were determined based on the accurate masses of the corresponding protonated molecules, their isotopic distributions, and the product ions’ *m*/*z* in MS/MS spectra. The tentative identification of the detected components was carried out on the basis of their elemental compositions, tandem mass spectra, and a search of the ChemSpider database and EMBL-EBI database (European Molecular Biology Laboratory’s European Bioinformatics Institute database) with a further selection of hits matching with the MS/MS data.

### 3.4. Determination of Moisture and Lipid Content

The content of moisture was determined by drying a sample at 105 °C until constant weight. All measurements were carried out in triplicate.

The total lipid content was determined using a Soxhlet apparatus with a connected reflux condenser. Microalga biomass (10 g) was extracted with methylene chloride for 6 h, after which the solvent was removed by evaporation using a vacuum evaporator (IKA RV 05 BASIC, Staufen, Germany), and the content of lipids was determined gravimetrically. The Soxhlet extraction was performed in triplicate.

### 3.5. Microbiological Analysis

Microbiological determination of the total aerobic microbial count, total yeast and mold count, *Enterobacteriaceae* and *Escherichia coli* was performed according to the ISO standard microbiological methods [58,59,60,61,62]. The spores of anaerobic mesophilic bacteria were determined on nutrient agar (HiMedia) incubated under anaerobic conditions at 30 °C for 48 h after 5 min in boiling water. Each test was performed in triplicate.

### 3.6. Determination of Metal Content

The chemical extraction for the determination of pseudo-total metal content was performed following the EPA method 3051A for solid phase [63]. For this, 0.5 g of solid phase was initially digested with the addition of 10 mL HNO_3_ and HCl (3:1) in the microwave unit of the Milestone Microwave Extraction System, Start E. After digestion, the extract was filtered into a 25 mL flask. The metal content in the digested samples was then determined using the ICP-MS technique (Agilent Technologies 7700 Series ICP-MS). All measurements were carried out in duplicate. The expanded uncertainty of measurement determined according to ISO 11352:2012 [64] was in the range of 20–30%.

## 4. Conclusions

Microalgae are undoubtedly valuable resources of numerous metabolites with important properties and applications. However, a precondition for the wider use of microalgae as a material for obtaining natural bioactive compounds is the development of a procedure for the isolation of compounds and the attainment of products. Using alternative solvents and innovative technologies can achieve a sustainable and efficient process for obtaining microalgal compounds and products, with optimal production costs and rational exploitation of the microalgal material.

It was established that the application of ScCO_2_ can have an important role as a pre-treatment, extraction process, and process for the reduction of microorganisms. Therefore, applying this green solvent can improve the exploitation of *T. obliquus* biomass and provide safe and clean products.

High-pressure pre-treatment applied prior to the ScCO_2_ extraction increased the yield of the lipophilic fraction, as well as the content of carotenoids, terpenes, steroids, and fatty acid derivatives in the supercritical extract. It was confirmed that after the ScCO_2_ extraction, the biomass can be used further as a potential source of bioactive compounds in various applications.

## Figures and Tables

**Table 1:** Chemical profile of supercritical CO_2_ extracts (control and pre-treated) of *Tetradesmus obliquus* biomass.

Compound	Structure	t_R_ (min)	Monoisotopic Mass	[M + H]^+^	Mass Difference (ppm)	Area (Counts)	
						Control	Pre-Treatment
**Pigments**							
Pheophytin *a*	C_55_H_74_N_4_O_5_	20.2	870.56592	871.5732	3.9	23,149	13,838
Vaucheriaxanthin	C_40_H_58_O_4_	14.0	602.433533	603.44079	5.2	72,962	70,087
Echinenone	C_40_H_54_O	15.2	550.417470	551.42474	4.0	10,175	31,354
Myxol 2’-fucoside	C_46_H_66_O_7_	19.1	730.480835	731.48813	2.5	72,962	14,927
Phytoene epoxide	C_40_H_64_O	20.2	560.495728	561.50299	2.5	72,962	138,351
**Fatty Acid Derivatives**							
Palmitamide	C_16_H_33_NO	13.8	255.25621	256.26349	2.4	816,302	1,062,902
2,3-Dihydroxypropyl palmitate	C_19_H_38_O_4_	14.2	330.27701	331.28429	1.9	18,191	19,599
Oleamide	C_18_H_35_NO	14.2	281.27185	282.27914	1.7	11,626,747	13,605,089
1,3-Dihydroxy-2-propanyl 5,8,11,14-icosatetraenoate	C_23_H_38_O_4_	14.5	378.27701	379.28429	0.6	35,250	10,435
Erucamide	C_22_H_43_NO	16.0	337.33447	338.34174	2.0	51,790	91,297
3-Hydroxy-1,2-propanediyl bis(9-octadecenoate)	C_39_H_72_O_5_	19.4	620.53798	621.54525	3.6	184,023	43,933
3-Phorbinepropanoic acid, 9-acetyl-14-ethylidene-13,14-dihydro-21-(methoxycarbonyl)-4,8,13,18-tetramethyl-20-oxo-, 3,7,11,15-tetramethyl-2-hexadecen-1-yl ester	C_55_H_74_N_4_O_6_	20.0	886.56085	887.56811	3.3	11,791	1,978,013
Methyl (3*R*,10*Z*,14*Z*,20*Z*,22*S*,23*S*)-12-ethyl-3-hydroxy-13,18,22,27-tetramethyl-5-oxo-23-(3-oxo-3-{[(2*E*,7*R*,11*R*)-3,7,11,15-tetramethyl-2-hexadecen-1-yl]oxy}propyl)-17-vinyl-4-oxa-8,24,25,26-tetraazahexacycl; o[19.2.1.16,9.111,14.116,19.02,7]heptacosa-1(24),2(7),6(27),8,10,12,14,16,18,20-decaene-3-carboxylate	C_55_H_74_N_4_O_7_	20.0	902.55575	903.56303	3.6	16,412	193,151
**Triacylglycerols**							
Triacylglycerol 54:7	C_57_H_96_O_6_	21.5	876.72069 *	877.72797	3.5	2,529,250	82,339
Triacylglycerol 54:6	C_57_H_98_O_6_	21.9	878.73634	879.74362	2.1	7,576,059	99,112
Triacylglycerol 54:4	C_57_H_102_O_6_	22.0	882.76764	883.77492	2.4	3,323,899	93,081
Triacylglycerol 54:5	C_57_H_100_O_6_	22.4	880.75199	881.75927	1.2	8,851,390	182,825
Triacylglycerol 54:3	C_57_H_104_O_6_	22.5	884.78329	885.79057	2.1	5,891,554	8953
**Diacylglycerophosphocholines and Diacylglycerophosphoserines**							
Phosphatidylcholine 33:2	C_41_H_78_NO_8_P	17.4	743.54651	744.55378	1.7	10,998	11,217
Phosphatidylserine 40:2	C_46_H_86_NO_10_P	18.1	843.59893	844.60621	1.0	37,610	
Phosphatidylcholine 38:3	C_46_H_86_NO_8_P	19.7	811.60911	812.61638	0.6	132,581	
Phosphatidylcholine 37:2	C_45_H_86_NO_8_P	19.9	799.60911	800.61638	1.4	100,848	4865
Phosphatidylcholine 38:2	C_46_H_88_NO_8_P	20.0	813.62476	814.63203	1.5	570,968	25,582
**Terpenes and Steroids**							
Loliolide	C_11_H_16_O_3_	6.4	196.10994	197.11722	0.1	173,169	506,025
Isoamijiol oxidation product	C_20_H_30_O_2_	14.9	302.22458	303.23186	0.5	122,024	452,509
Isoamijiol	C_20_H_32_O_2_	15.5	304.24023	305.24751	2.0	722,108	1,698,141
(3β)-3-Hydroxystigmast-5-en-7-one	C_29_H_48_O_2_	17.5	428.36543	429.37271	3.5	53,409	195,727

Note: The analysis was performed using UHPLC-ESI-HRMS. The separation of the compounds was achieved on the Acquity UPLC BEH Phenyl-Hexyl, 2.1 mm × 100 mm, particle size 1.7 m. The method is described in Section 3.3 in detail.

**Table 2:** Microbiological profile of *Tetradesmus obliquus* biomass: initial biomass and biomass after CO_2_ supercritical extraction (ScCO_2_-spent biomass). Results are expressed in colony-forming units (cfu) per g (initial and spent biomass).

Sample	Number of Microorganisms	Molds and Yeasts	Enterobacteriaceae	*Escherichia coli*	Spores of Anaerobic Bacteria
Initial biomass	910 × 10^4^	<10	49 × 10^3^	<40	240 × 10^2^
ScCO_2_-spent biomass	310 × 10^4^	<10	<10	<10	76 × 10^2^

**Table 3:** Metal composition of *Tetradesmus obliquus* biomass: initial biomass and biomass after supercritical CO_2_ extraction (ScCO_2_-spent biomass).

Metal	Initial Biomass (mg/kg)	ScCO_2_-Spent Biomass (mg/kg)
Cr	2.67	2.72
Mn	1070	1050
Fe	2510	3530
Co	1.66	1.64
Ni	2.63	2.82
Cu	39.4	38.0
Zn	149	150
As	<0.1	<0.1
Cd	0.083	0.080
Pb	5.50	5.36

## Data Availability

Not applicable.

## Supplementary Materials

The following supporting information can be downloaded at: https://www.mdpi.com/article/10.3390/molecules27123883/s1, Table S1: Kinetics of supercritical CO_2_ extraction (pressure 300 bar, temperature 40 °C) of *Tetradesmus obliquus* biomass. Extraction yield was expressed as % (*w*/*w* dry weight) described in Section 3.2. in detail.
