# Supplementary material for: Supercritical CO2 Extract from Microalga Tetradesmus obliquus: The Effect of High-Pressure Pre-Treatment
Source: Molecules. 2022 Jun 17;27(12):3883. doi: 10.3390/molecules27123883 (PMC9231020; doi:10.3390/molecules27123883)
Supplement: Supplementary file 1 [file molecules-27-03883-s001.zip › molecules-1735637-supplementary.pdf]

Table S1. Kinetics of supercritical CO_2 extraction (pressure 300 bar, temperature 40 °C) of *Tetradesmus obliquus* biomass. Extraction yield was expressed as % (w/w dry weight) described in Section 3.2. in detail.

**Extraction time (h)**	**Control**	**Pretreatment**
0.5	0.05 ± 0.01	0.19 ± 0.02
1	0.14 ± 0.03	0.33 ± 0.06
2	0.25 ± 0.02	0.64 ± 0.08
3	0.29 ± 0.03	0.87 ± 0.04
4	0.31 ± 0.02	0.92 ± 0.02
